# Utilization of paediatric isolation facilities in a TB-endemic setting

**DOI:** 10.1186/2047-2994-4-S1-P261

**Published:** 2015-06-16

**Authors:** A Dramowski, M Cotton, A Whitelaw

**Affiliations:** 1 Paediatrics and Child Health, Stellenbosch University, Cape Town, South Africa; 2 Medical Microbiology, Stellenbosch University, Cape Town, South Africa

## Introduction

In hospital settings, patient isolation is used to limit transmission of certain pathogens (e.g. *M. tuberculosis*, antibiotic-resistant bacteria and viruses causing respiratory and enteric infection). Data on the utilization of paediatric isolation facilities in low resource, TB-endemic settings is lacking.

## Objectives

To evaluate and make recommendations for utilization of isolation facilities in a paediatric department in a TB-endemic setting.

## Methods

Prospective daily observation of 18 paediatric isolation rooms at Tygerberg Children’s Hospital, Cape Town, South Africa, was conducted between 1 May 2014 and 31 October 2014 documenting: occupancy rate; indication for isolation; duration of isolation; application of transmission-based precautions and infection prevention (IPC) behaviour of personnel. Potential under-utilization of isolation rooms was determined by cross-referencing isolation room patient data with laboratory isolates of antibiotic-resistant bacteria, viral pathogens and *M. tuberculosis*.

## Results

Isolation room occupancy was 66% (2172/3294 occupied bed days), but varied significantly by month. Of 335 isolation episodes, 260 (78%) were for infection control purposes, including tuberculosis (130/260; 50%) and suspected viral infections (75/260; 29%). Children (median age 17 months; IQR 6-50 months) spent a median of 4 days (IQR 2-8 days) in isolation. During 1223 weekday visits to isolation rooms used for IPC purposes: alcohol-based handrub was available (89%); transmission-based precautions were appropriately implemented (71%); and personal protective equipment was provided (74%). Of 358 observed interactions between personnel and isolated patients, hand hygiene compliance was 65% and adherence to transmission-based precautions was 58%. Laboratory data identified an additional 135 patients with pathogens that warranted admission to an isolation room. Forty patients with 171 patient days of inappropriate isolation were identified.

## Conclusion

Children’s wards in settings with high infectious disease burden require isolation facility admission guidelines and regular review of utilization patterns and practices to ensure appropriate usage of this scarce resource.

## Disclosure of interest

None declared.

